# Easier and Faster Is Not Always Better: Grounded Theory of the Impact of Large-Scale System Transformation on the Clinical Work of Emergency Medicine Nurses and Physicians

**DOI:** 10.2196/11013

**Published:** 2018-12-13

**Authors:** Elaine Zibrowski, Lisa Shepherd, Kamran Sedig, Richard Booth, Candace Gibson

**Affiliations:** 1 Health Information Science Faculty of Information & Media Sciences University of Western Ontario London, ON Canada; 2 Division of Emergency Medicine Department of Medicine, Schulich School of Medicine & Dentistry University of Western Ontario London, ON Canada; 3 Department of Computer Science University of Western Ontario London, ON Canada; 4 Faculty of Information & Media Sciences University of Western Ontario London, ON Canada; 5 Arthur Labatt Family School of Nursing University of Western Ontario London, ON Canada; 6 Pathology & Laboratory Medicine Schulich School of Medicine & Dentistry University of Western Ontario London, ON Canada

**Keywords:** health care, emergency medicine, grounded theory, workflow, hospital

## Abstract

**Background:**

The effectiveness of Lean Thinking as a quality improvement method for health care has been contested due, in part, to our limited contextual understanding of how it affects the working conditions and clinical workflow of nurses and physicians. Although there are some initial indications, arising from prevalence surveys and interviews, that Lean may intensify work performed within medical environments, the evidence base still requires detailed descriptions of the changes that were actually introduced to individuals’ clinical workflow and how these changes impacted health care professionals.

**Objective:**

The aim of this study was to explore ways in which a Lean intervention may impact the clinical work of emergency medicine nurses and physicians.

**Methods:**

We used a realist grounded theory approach to explore the clinical work of nurses and physicians practicing in 2 emergency medicine departments from a single teaching hospital in Canada. The hospital has 1000 beds with 128,000 emergency department (ED) visits annually. In 2013, both sites began a large-scale, Lean-driven system transformation of their practice environments. In-person interviews were iteratively conducted with health care professionals from July to December 2017. Information from transcripts was coded into categories and compared with existing codes. With repeated review of transcripts and evolving coding, we organized categories into themes. Data collection continued to theoretical sufficiency.

**Results:**

A total of 15 emergency medicine nurses and 5 physicians were interviewed. Of these, 18 individuals had practiced for at least 10 years. Our grounded theory involved 3 themes: (1) organization of our clinical work, (2) pushed pace in the front cell, and (3) the toll this all takes on us. Although the intervention was supposed to make the EDs work easier, faster, and better, the participants in our study indicated that the changes made had the opposite impact. Nurses and physicians described ways in which the reconfigured EDs disrupted their established practice routines and resulted in the intensification of their work. Participants also identified indications of deskilling of nurses’ work and how the new push-forward model of patient care had detrimental impacts on their physical, cognitive, and emotional well-being.

**Conclusions:**

To our knowledge, this is the first study to describe the impact of Lean health care on the working conditions and actual work of emergency medicine nurses and physicians. We theorize that rather than support health care professionals in their management of the complexities that characterize emergency medicine, the physical and process-based changes introduced by the Lean intervention acted to further complicate their working environment. We have illuminated some unintended consequences associated with accelerating patient flow on the clinical workflow and perceived well-being of health care professionals. We identify some areas for reconsideration by the departments and put forward ideas for future research.

## Introduction

### Background

One outcome of encouraging health care systems to consider interdisciplinary approaches has been the overhaul of patient care environments with the use of the Lean Principles model. Lean Principles (commonly referred to as Lean Thinking or Lean) is a continuous method of process improvement pioneered by Toyota Motor Company for their car manufacturing production lines [1-6]. In brief, Lean is a customer-driven, continuous method of process improvement that asks an organization to focus on and reconsider how they are delivering what is of value to their customers [1-3,5]. Value is determined not only by what customers desire but also how fast what they desire is delivered to them [2]. Activities that are not contributing to value are considered to be wasteful in time and motion, and therefore, they are to be removed [1,2]. In contrast to other process improvement strategies, Lean is a bottom-up approach that relies on the input and engagement of both management and workers [1,3].

Although the state of the discourse on Lean in health care has been described as being relatively new [5], a systematic review by Moraros et al [6] concluded that the current evidence base is not strong enough to support upholding Lean as an effective quality improvement method for health care. Among the reasons underlying this assertion is that we have limited, contextual understanding of how Lean affects the multitude of internal and external variables [6] that exist within any health care setting.

Holden [4] and Rees and Gauld [7] advocated that efforts to enhance our contextual understanding must include exploration of the impacts of Lean-driven intervention on the working conditions and the actual work of individuals who are involved in the delivery of health care. There are some initial indications that Lean can intensify work performed within medical environments. Work intensification manifests under expectations that employees expend greater work effort by spending more time working, take on greater responsibility and/or more duties, or cope with fewer staff [7-9]. These pressures, in turn, can incubate increased levels of job-related stress and strain [10].

As part of a multiple case study by Rees [11], managers, nurses, physicians, and other support workers were interviewed about their involvement in the implementation of Lean interventions conducted in 3 hospital-based emergency departments (EDs) in New Zealand and found that employees from 2 of the 3 sites experienced work intensification. Although details regarding the nature and scope of duties that were affected by these interventions were not presented, individuals attempted to manage their elevated workloads with strategies including prioritizing duties related to patient care and using unpaid time to complete their work. Two Canadian studies reported on the experiences of nurses and clinicians and also of managers, with the widespread implementation of Lean across the province of Saskatchewan. Although the specifics of the interventions were not described by these studies, a random survey of 1173 nurses found that 49.5% reported that they experienced heavier workloads and greater levels of stress (rate ratio=0.29, 95% CI 0.24-0.35) and 58.2% reported feeling less engaged and had weakened morale (rate ratio=0.30, 95% CI 0.25-0.36) after Lean-driven changes were introduced into their workplaces ([12]; data described by Moraros et al). Clinicians and managers who participated in the provincial implementation of Lean health care acknowledged, in hindsight, that interventions were overwhelming for their staff [13]. Hung et al surveyed 1333 health care professionals in the United States, including physicians and clinical support staff, before and after their ambulatory care clinic had undergone a Lean-based redesign of their clinical processes [9]. Although the details of interventions undertaken by individual clinics were not presented, these authors noted that Lean redesign included the composition of care teams and their workflow. The surveys probed aspects of worker engagement and teamwork, and participants were also asked to complete a measure of occupational burnout. After the redesign, nonsignificant increases were observed in both groups in terms of their scores on measures of engagement and work satisfaction. Despite these improvements, Lean changes did not appear to mitigate job-related stress as statistically significant increases in emotional exhaustion were reported by both groups (physicians parameter estimate=0.39, *P*<.01, clinical staff parameter estimate=0.365, *P*<.05 for nonclinical staff).

### Objectives

If we are to more fully advance our contextual understanding of Lean in health care, including how it may be linked to work intensification, we will need to disseminate more granular levels of description of the changes that were introduced to clinical activities within local settings and the impacts, both intended and not, these modifications have on the professionals who practice within that working environment. The purpose of this study was to explore the ways in which a Lean intervention may enhance or disrupt clinical work and within what contexts.

## Methods

### Study Design

We utilized a grounded theory approach with a realist lens. Pawson [14] contends that when we explore any intervention, we must attend to contexts in which it is situated. Context is not merely unwelcome noise nor a confounding variable to be controlled for [14]. Any context will have embedded within it socially interactive factors, including the individuals who are experiencing the intervention, their interpersonal relations, the institutional setting of the intervention, and the impact of its greater infrastructure. These factors will act to support or constrain how well an intervention is taken up in a given setting. In sum, Pawson [14] describes the realist mantra is one that attends to what works, for whom, and in what circumstances.

We selected grounded theory because it is a methodological approach that seeks to explore how persons experience and give meaning to events [15,16]. Rather than focus on testing of specific hypotheses or theories, grounded theory seeks to describe social processes from data that are systematically collected or grounded in their participants, and data are analyzed throughout the course of the study [15,16]. This methodology has been recognized to be particularly useful for exploring phenomena about which little is known [17,18].

### Hospital Sites and Participants

From July to December 2017, we recruited 20 emergency medicine professionals (15 nurses and 5 physicians) from 2 sites of a teaching hospital in Ontario, Canada. Eighteen of these individuals had been practicing emergency medicine for at least 10 years. The hospital has 1000 beds with 128,000 ED visits annually. The reported wait times for the hospital’s ED were among the worst for the province, and in response to this, in 2013, both sites began a large-scale, Lean-driven, system transformation of their practice environments.

### Data Collection

We recruited professionals using an email that was sent to the official, hospital accounts of emergency nurses and physicians by the ED on behalf of our team. To be eligible for participation in this study, a professional needed to have been practicing at the hospital for a minimum of 1 year, beginning no later than a specified date which preceded the ED’s planning for the Lean transformation. Nurses and physicians were asked to directly contact EMZ via her official, university email account. Interviews were arranged at a time/location convenient for the professional, and these meetings were audio-recorded for later transcription into verbatim, anonymized documents by a professional service. The department was not informed of participants’ identities. Interviews were scheduled for 1 hour, which is consistent with grounded theory [18]. Participants received a Can$ 20 gift card as an honorarium. Both university and hospital health research ethics boards approved the protocol for this study. Consistent with a realist focus, the interview guide probed the physical structure of the ED, organization of patient flow, individuals’ clinical workflow, opportunities for nurses and doctors to collaborate during patient care, and the impetus and planning around the transformation of the ED. Data collection was organized around a constant comparative process that hallmarks grounded theory [15,16,19].

### Data Analysis

After each interview, notes were written about dialogue with the professional, and the interview guide was refined to probe emerging ideas across successive participants. Once a transcript was received from the professional service, its accuracy to the original recording was reviewed. As the interviews proceeded, their transcripts were first coded into categories with the use of MAXQDA software (Version 11.2.5, VERBI Software, Sozialforschung GmbH, Berlin, Germany). We checked on the consistency of coding across 3 team members (EMZ, RB, and LS) for 2 of the transcripts. Coding continued alongside data collection so that new information was compared with existing codes. Through repeated review of the interview transcripts and our evolving coding, we organized categories into themes. Our data collection continued to theoretical saturation of meaning at which point we felt that the amount of information we gathered was sufficient to support our understanding of participants’ perspectives and that any additional interviews were not likely to introduce major modifications of our understanding of the data gathered in our study [20,21]. For our study, we sensed theoretical sufficiency after 20 interviews.

## Results

### Themes

The results of our study illuminated the impact of large-scale, system transformation on emergency medicine nurses and physicians with 3 themes: (1) organization of our clinical work, (2) pushed pace in the front cell, and (3) the toll it all takes on us. In the following sections, we describe the clinical practice environments of the ED both before and after their redesign and our 3 themes in greater detail. As is consistent with grounded theory, we have supplemented our results with anonymized, illustrative quotes from our participants [16]. Quotes with a generic identifier beginning with “N” are from an emergency nurse, whereas generic identifiers beginning with a “P” are from an emergency physician.

### The Clinical Practice Environments

#### Original Model

The original practice configuration of the ED involved a triage area that triaged patients to 3 pods (labeled A, B, and C). Pod A housed patients with the most acute care needs. Less ill patients were triaged to the other 2 pods. Patients requiring major resuscitation, mental health assessment, or special emergency procedures, such as an eye examination, were included in ED spaces outside the 3 pods.

Pod A was configured with 10 beds each spaced with surrounding curtains. A central desk with computers and a departmental, landline telephone was available for use by registered nurses and unit clerks, whereas physicians had a desk area off to 1 side of pod A. Medical supplies for all patient care areas were distributed from a central supply.

In terms of staffing, 2 nurses were assigned for triage duties, 3 to 4 nurses for pod A, and other nurses in the additional care areas. Aside from overnight hours, 3 emergency physicians attended to patients throughout the ED. Nurses worked in 12-hour shifts and physicians worked in 8-hour shifts. In the event that a nurse was called in for additional coverage in the ED, she/he would work 8 hours. Physicians working overnight in the ED were scheduled for a 6-hour shift. At the end of their shift, it was common for physicians to wrap up patient care on their own time.

#### Reconfigured Model

In 2013, both sites began an emergency department system transformation (EDST) involving both the reconstruction of their physical environment along with changes made to their patient care processes. The plans for the transformation were developed in collaboration with an international consultant with expertise in Lean health care, front-line staff, and management. The overall goal, and resulting byline, for the transformation was that it would make the ED easier, faster, and better. All patient care areas were reconceptualized into 3 bubbles or cells. Pod A became the front cell and it was split into 3 zones (blue, green, and orange). Each colored zone was equipped with 3 beds and 6 chairs. The physicians, nurses, and learners assigned to each colored zone were allocated portable, battery-operated, computer, workstations on wheels (WOW) clustered around their stretchers. The staff was encouraged to use the WOWs in a standing posture. The unit clerk was situated at a central hub that included a photocopier/fax/printer as well as a landline telephone. Portable phones were assigned to nurses and physicians in each zone. Although supplies were still provided from central supply areas, medical supplies stocked for each cell were reconfigured.

During each shift, the reconfigured ED was staffed with a total of 13 nurses (2 nurses at triage, 1 primary assessment nurse [PAN] and 2 nurses for each of the 5 patient areas across the cells) and 3 emergency doctors (1 assigned to each colored zones in the front cell). In addition, a communications clerk and ED technician would be working with these professionals. In terms of operating schedules, the 3 zones were opened during the day and evening and overnight with reduced staff working in 1 or 2 of the zones depending upon patient volumes and staffing. The number of scheduled hours for nurses’ and physicians’ shifts in the ED did not change.

### Organization of Our Clinical Work

#### Original Model: Physicians

In the original model, physicians explained that the ED was organized by patient acuity. At triage, an emergency nurse assigned a Canadian Triage Acuity Scale rating to every patient that categorized one’s medical priority to be seen [22]. A patient would be brought to their assigned bed by a nurse and would remain there until their point of disposition. Using a computerized boarding system, physicians selected or pulled patients specifically into their care. Physicians were not assigned to a particular pod within the ED, and they would move or float around to provide care.

Even during periods of high patient volume, physicians described that the original ED model allowed them to generate an overall, comfortable cadence of patient flow. This was primarily afforded through opportunities for physicians’ to make one or more strategic patient pulls during their shift. Interviewees explained that, during a given shift, they were able to review the ongoing list of triaged patients and use this list to make decisions regarding the type and number of patients they should pull into their care. By making some strategic patient pulls, doctors perceived that they were able to maximize their clinical efficiency:

We would just kind of do the sickest people first, it’d go to the sickest person, usually by triage code. And sometimes you would do, just for efficiency as well, so if there was a sick person and a not sick person in one of the three rooms, I would often grab two of them. Because one would be quick and one would be longer, but I’d only walk in there once as opposed to twice. What it also gave you the chance to, like, if you saw three sick people in a row and had a lot of things going on, the sensible thing to do is to see that twisted ankle, sew up the finger, in between, so that there’s kind of a self-driven load or control the amount. But it also allowed you to, you know, you know you’ve got 10 minutes so you can call out some of the quick ones and not at the expense of the others. So it was self-driven movement.

P201

Strategic patient pulls were also used to support the efficiency of other doctors, and participants described using strategies including pulling specific patients into their care so that another colleague was not caring for too many complex patients at one time, and as this participant explained, streamlining your cases to avoid issues at the time of handover for the next doctor coming on shift:

We’d always had an agreement in the last two hours of the shift, that you could clearly go ahead and pick out cases that you felt were likely to be simpler so that you would have to, less likely to hand over those cases. There’s really limited utility in seeing somebody 15 minutes before you’re supposed to leave. You’re just going to have to hand it over right to another person who is basically going to have to start over anyway.

P202

Moreover, doctors felt that because they were able to float across the 3 pods they were able to band together and support one another by covering for colleagues during their breaks and checking in on another doctor’s patient if they were already heading over to a particular pod:

You could say, “There’s that really urgent person that just came into bed 2, can you go see that person?” And we would work, the physicians who worked together would work as a team.

P204

#### Original Model: Nurses

In the original model, nurses explained that their work was organized by designated bed assignments, that is, during their shift, a nurse would be assigned to a specific block of beds within a pod and it was understood that:

Those were my patients regardless. And if I’m going on break, I have to make sure that there’s coverage for them, and if I have to leave the room. I’m primarily responsible for them.

N106

Nurses explained that the process of assigning them to bedsides held several advantages to the delivery of patient care. As a nurse was likely to be the first provider a patient encountered in a pod, she/he played a very important role in the critical assessment and monitoring of that individual. As 1 of the nurses explained:

It was good because you could see them from the beginning to the end. You could tell if treatments and interventions were making them better or not having any effect at all. If they’re coming in and they’re in their worst possible presentation, I need to know if what we have done has helped them. And if it’s not, then I need to report that to the physician so we could try something else because it’s not working.

N109

Second, both doctors and nurses asserted that nurses at bedsides often freed up physicians’ time, which, in turn, often allowed a doctor to be able to spend more time with other patients or to be able to pull more patients into their care during a shift:

The best part was the continuity of care. So when we’re assigned a bed (for the patient), that nurse stayed with them. There weren’t multiple handovers and you kind of knew where they were. You could plan your movements in the department knowing they were there. You had a consistent nurse assessing changes, physiologic changes, anything that came up was picked up, the orders were consistently carried out, and you didn’t have to worry about that.

P201

Furthermore, nurses viewed that being with patients throughout their trajectory meant that they had an important opportunity to establish rapport with patients and their families. Nurses were valuable in answering their questions, comforting them, and gaining information from family members that was relevant to the patient’s condition:

I find you had more time to speak with patients, the families, getting to know just some little nuances that could tip you off. You had the time to talk with them. I also found you had more time to build a relationship with your patients.

N110

Finally, nurses perceived that the original configuration afforded nurses working together in a pod to develop a strong sense of camaraderie. In pressing moments, nurses recalled uniting together to work as a team. A nurse recalled what it was like to practice in the pod A of the original model, which was used to treat the most urgent cases:

I liked it. I didn’t mind working in Pod A. It was nice, to have people around, to have people helping. Everybody would know what was going on, in a general sense, of all the patients in the Pod A area. Everyone else was right there that could come and help you deal with it at that time. If someone came in with a heart attack, per se, you had them with you the whole time.

N111

#### Reconfigured Model: Physicians

During their interviews, doctors perceived that their site had shifted from an acuity-based model to one that was orientated toward maximizing the number of patients their ED sees daily. One of the physicians summed up the new situation as:

Time management was very different than it is now. We are now in a push-forward model.

P200

The system transformation generated a new staff role in the ED, the PAN whose primary job is to direct patients into a colored zone of the front cell. Once a patient has been directed to one of these zones, the physician will assess the patient. Ideally, this will occur within a targeted period. In the event that a patient requires further assessment and/or treatment, they will be physically moved from the front cell to the middle and/or back areas. Although the patient is still cared for by the same physician after they are moved to another cell, the physician attending is required to begin working with a new set of nurses.

Physicians were frustrated about how the reconfigured model had decreased the level of control they had over their clinical workflow, and therefore, they had less ability now to control the cadence of the ED. Assigning physicians to particular zones of the ED also diminished their abilities to interact and support one another. Rather than be able to float from pod to pod*:*

In the new system, the physicians are like islands. We do not work with each other. We do in a very minimalistic fashion.

P203

They also sensed that their department expected more as they were, essentially, now required to see one-third of all the patients that were pushed forward from triage during their shift. An interviewee admitted:

It can be a very overwhelming system to work with because it basically puts all the pressure on you. So, if you are really tied up with someone who’s very ill or a very complex patient, then you are constantly, like “Oh my god, I’ve got these other patients that are mine that no one else is going to see them.

P202

Given that patient flow was delegated to the discretion of a PAN, interviewees noted that their ability to make strategic patient pulls was diminished, and as a doctor who was interviewed noted, the PAN did not always understand why an attending would want, or even request, that they not be given several complex patients within a short period:

In the old model, I had more choice over who I was going to see. You could allot your time easier and pick the patients you wanted to see. You don’t want a PAN nurse to give you five critically unwell patients in a row. You want them to put in a few easier ones to help you with your flow of patients and sometimes they don’t do that, they keep putting them in.

P200

Finally, physicians highlighted that being assigned to a particular zone did not mean that they would remain stationary during a shift. It was common for an attending to move back and forth, and even repeatedly so, within and between the front, middle, and back cells. A variety of examples were given of why they needed to do this including moving back and forth between front and middle cells to check on several patients, needing to retrieve medical supplies, changing out a dead battery on a WOW, and needing to move a patient out from a chair in the front cell so that they could speak with the individual in a more private manner.

As a participant explained, some doctors perceived that the reconfigured model had diminished the overall role of the physician in the ED because:

Emergency physicians are used to multi-tasking. We’re used to a busy environment. We’re used to an unpredictable environment. But what we’re not used to is not having control with regard to how we manage our environment. And that is the salient difference. It’s taken the complete autonomy and leadership quality that a physician provides in the emergency department completely out. So now we come to work and you’re just assigned a little zone and a little box and you’re told what to do.

P203

#### Reconfigured Model: Nurses

Nurses also perceived that the reconfigured ED diminished their opportunities for collaboration. First, the new configuration relies on fewer nurses to provide care, and if fully staffed, there are 2 nurses working within a cell. However, as participants explained, in situations such as when a nurse calls in sick for their shift, the individual may not be replaced by another colleague:

So, yeah, sometimes there are two nurses, but a lot of times, particularly on nights, there’s now one. Sick calls have gone through the roof, so, like, Saturday night they were five nurses short, last night there was three. So we find ourselves working with one nurse.

P201

Second, nurses noted that aside from times of patient handover, there could be little, if any, interaction among the nurses practicing in other cells:

You interact very differently because now you are assigned to a cell. You’re focusing on the cell. You’re not focusing on if you’re one cell and just the way the cells are. Your back is turned towards one cell and you don’t know what they’re doing, you don’t know if they need help. But you can’t help them either because you’re working at a cell and you might have one to two doctors, you might have residents and if you’re short staffed you’re now working in the cell by yourself.

N103

Opinions were split amongst nurses and physicians about the impact of the reconfigured ED on the quality of nurse-doctor interaction. Some doctors felt they had better opportunities to establish a working rapport with nurses in the new model, whereas others expressed they worked better with nurses in the original configuration. Although nurses generally acknowledged that it was easier to keep track of an attending in the reconfigured ED, it did not necessarily mean that you would be working collaboratively with them.

Some nurses felt that the reconfigured ED increased the power differential between nurses and doctors:

It now means that it’s one physician, he’s like, “Dah, dah, dah,” so now you’re his robot. “Do this, do this, I need that, you need to go give that, you need to do this.”

N108

Nurses asserted that, by pushing all patients through the front cell, the new configuration had fundamentally changed the nature of their duties. Nurses working in the front often carried heavier workloads, involving more physical work. As this interviewee explained:

I would say work for nurses, to give you an idea, in the new model, where most of the blood work, IVs and everything else is all done in the front bubble. Every patient is seen in the front bubble. And I’m not saying that middle and back bubbles are easy to work, but at the same time, I wouldn’t say you’re doing as much work in those areas. So, physical work-wise, definitely there’s a lot more imbalance. I would say that would be the main thing, is that, in the older system, there was a lot more equalization.

N114

Moreover, nurses viewed that the redistribution of physical work to the front cell, in turn, diminished the purpose of a registered nurse in the reconfigured ED away from being a key actor involved in ensuring continuity of care.

### Pushed Pace in the Front Cell

Although interviewees noted there were times when the reconfigured ED worked well to meet patient demands, there were times that both sites struggled with high patient volumes:

Some days I feel like there’s a bus that drops them all off at the same time. That’s what it feels like. It’s every day. It’s not weekends. It’s every day.

N112

Doctors and nurses viewed factors that were contributing to ongoing patient volume pressures included the sites receiving greater numbers of complex cases including those transferred from smaller communities along with increased demand for mental health and addictions treatment:

Acuity-wise, I am finding patients are sicker, in general. There are fewer beds [everywhere], so people are sicker before they come into the hospital, and also just the sheer numbers. We are averaging 200 to 230 patients in 24 hours.

N110

During times of high patient volume, interviewees were aware that the reconfigured ED model emphasized flowing them through:

We’ve got to get people moving. We’ve got to do this. We’ve got to do that. There’s push from all over. There’s push from the physicians in the front. There’s push from management. There’s push from PAN or charge nurse, either one. Keep it moving. Keep it moving.

N113

The front cell was identified as the primary area where professionals experienced the brunt of the impact of high patient volume. Although a PAN was viewed as being involved in the ongoing flowing of patients, some interviewees perceived the role as being one that did not require the same skill set as the other registered nurses in the ED:

[Role of a PAN] Is to push them and to keep them going and keep the flow. One of our co-workers said, “a monkey could do that job.”

N109

The PAN nurses, they call them primary assessment, but they don’t really do it. It’s us, but they’re the ones who are pushing.

N108

Several participants recalled incidents during which they served as a PAN in the front or they interacted with one that involved tension with other staff:

For me, I like to go and talk to everyone face-to-face. And I’ll say, “I’m PAN nurse today.” And some people roll their eyes because I’m a mover, organizer, shaker, and I do the rob Peter to pay Paul. I’ll move and shuffle people like a Jenga.

N112

Push the pace. And you’ll say to the PAN nurse, “Can you just give my zone a 10-minute reprieve? I have a bunch of reassessments to do and then I really need to go eat something.” And they’ll still fill your beds up because they were told by management that they needed to continue to fill beds up.

P204

Professionals perceived that a crowded ED amplified the challenges that the new configuration already introduced to their clinical work. First, there were capacity issues associated with flowing all patients through the front. As a nurse who was interviewed counted, the front cell typically contained a minimum number of people that would need to be working within that space:

You used to have, you know [in the old model], if you had three physicians on, there might be two people seeing a patient in Pod B, and there might have been one doctor seeing a patient in Pod C. There might not have been anyone in Pod A, which is where the front bubble is now. But everybody now, there is one doctor per each cell they could have upwards to three learners. If the two nurses are there, which is great, there are two nurses, so that could be five to six people per area. So you’re upwards to 18 to 20 people before you’re even involving the patients, in that area.

N105

Add to this mix, patients and any family members that may have accompanied them to the ED and the front became very congested:

It’s like a hornet’s nest. It’s the best way to describe it.

N110

As the ED filled, so did the need to keep moving patients around the ED. Interviewees asserted that figuring out where to move patients could be complex and time-consuming:

We’re always behind. We can’t keep up and whereas, previously, we really could. So, it’s like this constant Rubik’s Cube. Like, move this person here and move that person there. And it’s like never-ending. You could be moved around several times because of the fact that there is somebody else competing for your stretcher who is iller than you. And then, it’s eventually deemed, okay, you can’t have a stretcher anymore, you’ve got to sit in a chair.

P202

Participants recalled being interrupted more, struggling to keep up with what needed to be done for their patients’ care, and often feeling overwhelmed while working. In a crowded front cell, some nurses also admitted that their clinical workflow could become very fragmented to the point that they could not complete everything to the standard they desired:

What happens often too I find is that there is a lot of pressure to get these people in and be seen that they just bring them all in. Charts get disorganized. There’s no kind of methodical movement to all of this stuff because “Oh, this person needs this and that.” They may need that done, but the policy procedure as far as nurses go, they need vitals after. They might need to be fully disrobed. You need to listen to a chest. There are all these little bits that have to occur based on standards of care that don’t always happen in this environment because of the movement of people so quickly.

N103

On a similar note, some physicians recalled moments in the front when they needed to be more vigilant about what nurses were doing (and not doing). During times when they sensed a nurse could actually miss an order, they needed to make an effort to verbally push that nurse more to ensure that the work was actually carried out. As a doctor explained:

I’ve had to change my practice in the bubble to say, “Do not move that person until this, this, and this are done.” Because if I don’t do that I will go to a room two hours later, three hours later, and things aren’t done.

P203

Participants noted that during periods of high volume, eventually, patient movement would stop due to bottlenecks in the front cell or the hospital had become bed-blocked, meaning that the number of patients requiring admission had exceeded the number of beds that were available.

### The Toll This All Takes On Us

Participants admitted that working in the front cell was often a stressful experience that impacted them physically, cognitively, and emotionally:

We are in an area where it is so high stress that sometimes...last night we had a [complaint anonymized] case come in. I’ve been there for [number anonymized] years and I felt like I was going to have a panic attack. That’s the kind of environment. It is stressful, stressful, stressful.

N114

I just find most shifts I just keep my head above water. Like, you feel like you’re drowning constantly.

N105

Interviewees identified several conditions of their working environment including the constant movement required from doctors and nurses in the front cell during patient care, difficulty finding the time and place to take a nutrition break during a shift, and being required to stand for long periods often resulted in doctors and nurses feeling very physically fatigued:

Our legs are tired. Every nurse, guys and girls alike, even the docs, we’re all wearing the compression stockings. Before we had chairs where we could sit down and chart. Now we’re standing up at the computer doing our charting. You’re standing your full 12 hours.

N112

Professionals also recalled moments where they felt cognitively overextended. During these times, they described having difficulty maintaining attention, needing information to be repeated to them, forgetting patient names, second-guessing whether they had completed a task fully (or not), and using moments where they used a more menial task, such as retrieving supplies, as an opportunity to take a cognitive respite. Moreover, some interviewees admitted that to try to cognitively decompress after working a shift in the ED, they needed to be socially isolated for some period from family and friends:

It’s just sensory overload. You're constantly, in the front bubble, you're constantly being pushed to get patients in, get patients out, get patients in, and get patients out. For me, and this doesn’t happen all the time, so I don’t want to paint a bad picture, but I shouldn’t go home so mentally tired that I don’t want to socialize with people.

N102

Some days you physically feel fine, but, mentally, you are drained. And it’s because you have ninety patients’ information running through your mind.

N101

Most interviewees recalled incidents where they had been on the receiving end or witnessed moments of pushback from patients to staff (and vice versa). These incidents were difficult to experience and witness, and most times, these events seemed to catalyze from patients’ frustration with wait times:

We [the general public] don’t seem to control our tempers anymore. We [the general public] don’t seem to control our outlets. We [the general public] want instant gratification, we [the general public] want this and they get angry and they feel it’s acceptable to become angry, yelling, threatening to hit. Lives have been threatened in the emerg. You hear some events that have happened and the nurses are becoming angry at the patients as well.

N108

Overall, participants sensed that colleagues’ morale had declined at work and, as evidenced by the following statements, showing awareness of colleagues that were contemplating leaving their position at their site or had recently quit their job:

Of the heavily trained people, the people that I perceive as the strongest up-and-comers, a lot of them are peeling off.

P201

I don’t know where it’s heading, but I just know that something has to change because we’re going to lose more. At least, from a nursing aspect, we’re going to lose more. I have been in this department for (number anonymized) years. I love emergency medicine but I hate what is happening. Five years ago, I wouldn’t have even looked at the job board to get out.

N110

## Discussion

### Principal Findings

Emergency medicine is a highly complex medical discipline characterized by fast pace, interruptions, multitasking, overcrowding, and unpredictability [23-29]. Although the EDST was supposed to make the ED work easier, faster, and better, the participants in our study described that the Lean-driven changes made to their practice environment, most especially with the design of the front cell, had the opposite impact.

Physicians and nurses spoke about how assigning them to work within the front cell fundamentally disrupted routine patterns of how they interacted with patients and with each other. Doctors noted that despite the responsibility they held within the reconfigured ED, they had diminished autonomy over their work. The physicians in our study found it especially disruptive to have reduced opportunities over the course of their shift to plan and execute as many strategic patient pulls as they judged necessary. This should not be surprising given that Kovacs and Croskerry [23] posited that the most important type of information used by emergency physicians in their clinical decision making relates to their patients’ acuity, and moreover, that Schubert et al identified time management as one of the defining features [29] that distinguishes expert emergency physicians from novices. By limiting their ability to make strategic patient pulls, the ED was unintentionally disrupting physicians’ ability to exercise their professional expertise. Therefore, any moments of tension between PAN and attending, where a physician requests that patient flow be slowed (or even halted), are very likely important signals of physicians’ heightened situational awareness. Moulton et al [30-32] observed that surgeons often experience transitional moments during patient care when they feel the need to slow down. These transitions may be routine or unplanned and can result from factors including recognition of the need to deal with distractions and sensing one’s fatigue. Moulton asserts that slowing down is the “crucial part of expert surgical judgment, and failing to transition during critical moments may lead to medical error and patient harm” [31]. Given that physicians recalled moments where they felt they needed to slow down patient flow for reasons similar to that observed by Moulton, rather than continuing to push the pace, we suggest that the ED should reframe these requests as important opportunities for assessment of potential risks. Future exploration of the potential relationship of emergency physicians’ strategic patient pulling and requests to slow down patient flow with expert physicians’ judgment and distributed cognition is warranted.

Although a nurse may still be the first provider whom a patient encountered within the front cell, the quality of that nurse-patient interaction may have shifted significantly. The nurses who participated in our study did not indicate that their department had intentionally restricted their involvement in certain clinical activities, but they did perceive that they held diminished value within their department after the reconfiguration of the ED. Nurses viewed that the front cell required less use of their critical assessment skills, they were less involved in monitoring patients, were being pushed toward carrying out more general tasks that often involved physical work, and they had fewer opportunities to develop a rapport with patients and their families. Although some physicians perceived their working relationship with nurses had improved after the reconfiguration, nurses did not share this opinion. Nurses felt they had fewer opportunities to collaborate with physicians and, compared with the original model, they were now working less with physicians and more for them. In the United Kingdom, intentional narrowing and standardization of workers’ duties under Lean has been associated with deskilling of taxation civil servants [33] and automotive manufacturing employees [34]. We found that the PAN, a role that was directly borne out of the EDST, was viewed by some professionals as being a position that did not draw on the same skill set as required by other registered nurses within the ED. This observation taken together with other above-mentioned perceptions of nurses’ work suggests that some unintentional deskilling may have been introduced in the ED with Lean. As such, the relationship between clinical workflow redesign and deskilling of nurses requires further attention.

An argument can be made that as emergency medicine is highly complex, by definition, the clinical work performed by its nurses and doctors will always be intense. That being said, the acceleration of patient flow to the front cell appeared to further ramp-up the existing pressures faced by health care professionals in the ED. The participants in our study described how several years after their department underwent a Lean redesign, their clinical workloads were intensified. They reported greater pressure to keep patients flowing, spent time moving patients around the front cell, were more likely to be interrupted while working, carried out more menial tasks that added to their workload, and were not always confident that their work was completed to the desired standard. They also admitted feeling emotionally and physically exhausted, noted more of their colleagues requested sick time away from work, were aware of incidents of tension between colleagues and patients, and knew that other professionals had already or were contemplating leaving their jobs. It has been estimated that at least 60% of emergency medicine physicians and nurses have experienced symptoms of burnout syndrome [35-37]. Although we are unable to estimate the prevalence of symptoms in our study, the ways in which our participants described how their work impacted them physically, cognitively, and emotionally suggest that they are at risk for developing burnout syndrome. Similar to the results of Hung et al, we did not find that Lean redesign mitigated levels of job-related stress perceived by nurses and physicians [9]. Unlike these authors, we did not find that our participants were more engaged and more satisfied with their work after the reconfiguration of their practice environment. Our findings suggest that the ED revisits and re-evaluates its Lean-informed design of the front cell including its relationship with work intensification, workplace stress, and worker burnout.

Lean has been described as a quality improvement approach that depends on worker engagement and input [3,4]. Although in this study we have not addressed our participants’ conceptual understanding of Lean or their involvement in the planning and implementation of the ED’s reconfiguration, we did not sense any unwillingness from them to try to ensure that the intervention was successful for their department and hospital. Rather, despite the ergonomic challenges they faced, our interviewees seemed to be quite passionate about their work and commitment to patient care. It is unclear, at present, how the perceptions of the nurses and physicians who deliver patient care in the reconfigured ED resonate and align with what hospital management expected the intervention would achieve. Future exploration of what constitutes success in Lean-driven health care is warranted.

### Limitations

As our study involved 2 sites of a single teaching hospital, its findings are representative of our local context. Further research into the impact of Lean health care on the clinical work of nurses and physicians practicing in other emergency medicine departments and in other medical settings is necessary to explore the transferability and resonance of our findings.

### Conclusions and Implications

To our knowledge, this study is the first grounded theory regarding the impact of Lean on the working conditions and actual work of emergency nurses and physicians. We theorize that rather than support health care professionals in their management of the complexities that characterize emergency medicine, the physical and process-based changes introduced by the Lean intervention acted to further complicate the environment under which they delivered patient care. Our research has illuminated some unintended consequences associated with accelerating patient flow on the clinical workflow and perceived well-being of health care professionals. Nurses and physicians described several ways in which the new model disrupted their established practice routines and resulted in the intensification of their clinical work. Participants also identified indications of the deskilling of nurses’ work and how the new, push-forward model of patient care had detrimental impacts on their physical, cognitive, and emotional well-being. On the basis of our findings, we advocate for future exploration of the relationships between emergency physicians’ use of strategic patient pulls and requests to slow down patient flow with expert physicians’ judgment and distributed cognition, clinical workflow redesign, work intensification and deskilling, and Lean health care and burnout symptoms experienced by nurses and physicians.

## Abbreviations

ED: emergency department
EDST: emergency department system transformation
PAN: primary assessment nurse
WOW: workstations on wheels
